# A panel of CSF proteins separates genetic frontotemporal dementia from presymptomatic mutation carriers: a GENFI study

**DOI:** 10.1186/s13024-021-00499-4

**Published:** 2021-11-27

**Authors:** Sofia Bergström, Linn Öijerstedt, Julia Remnestål, Jennie Olofsson, Abbe Ullgren, Harro Seelaar, John C. van Swieten, Matthis Synofzik, Raquel Sanchez-Valle, Fermin Moreno, Elizabeth Finger, Mario Masellis, Carmela Tartaglia, Rik Vandenberghe, Robert Laforce, Daniela Galimberti, Barbara Borroni, Chris R. Butler, Alexander Gerhard, Simon Ducharme, Jonathan D. Rohrer, Anna Månberg, Caroline Graff, Peter Nilsson, on behalf of the Genetic Frontotemporal Dementia Initiative (GENFI), Lize Jiskoot, James B. Rowe, Alexandre de Mendonça, Fabrizio Tagliavini, Isabel Santana, Isabelle Le Ber, Johannes Levin, Adrian Danek, Markus Otto, Giovanni Frisoni, Roberta Ghidoni, Sandro Sorbi, Florence Pasquier, Vesna Jelic, Christin Andersson, Sónia Afonso, Maria Rosario Almeida, Sarah Anderl-Straub, Anna Antonell, Silvana Archetti, Andrea Arighi, Mircea Balasa, Myriam Barandiaran, Nuria Bargalló, Robart Bartha, Benjamin Bender, Alberto Benussi, Luisa Benussi, Valentina Bessi, Giuliano Binetti, Sandra Black, Martina Bocchetta, Sergi Borrego-Ecija, Jose Bras, Rose Bruffaerts, Marta Cañada, Valentina Cantoni, Paola Caroppo, David Cash, Miguel Castelo-Branco, Rhian Convery, Thomas Cope, Giuseppe Di Fede, Alina Díez, Diana Duro, Chiara Fenoglio, Camilla Ferrari, Catarina B. Ferreira, Nick Fox, Morris Freedman, Giorgio Fumagalli, Alazne Gabilondo, Roberto Gasparotti, Serge Gauthier, Stefano Gazzina, Giorgio Giaccone, Ana Gorostidi, Caroline Greaves, Rita Guerreiro, Carolin Heller, Tobias Hoegen, Begoña Indakoetxea, Lize Jiskoot, Hans-Otto Karnath, Ron Keren, Tobias Langheinrich, Maria João Leitão, Albert Lladó, Gemma Lombardi, Sandra Loosli, Carolina Maruta, Simon Mead, Lieke Meeter, Gabriel Miltenberger, Rick van Minkelen, Sara Mitchell, Katrina Moore, Benedetta Nacmias, Jennifer Nicholas, Jaume Olives, Sebastien Ourselin, Alessandro Padovani, Jessica Panman, Janne M. Papma, Georgia Peakman, Michela Pievani, Yolande Pijnenburg, Cristina Polito, Enrico Premi, Sara Prioni, Catharina Prix, Rosa Rademakers, Veronica Redaelli, Tim Rittman, Ekaterina Rogaeva, Pedro Rosa-Neto, Giacomina Rossi, Martin Rosser, Beatriz Santiago, Elio Scarpini, Sonja Schönecker, Elisa Semler, Rachelle Shafei, Christen Shoesmith, Miguel Tábuas-Pereira, Mikel Tainta, Ricardo Taipa, David Tang-Wai, David L. Thomas, Paul Thompson, Håkan Thonberg, Carolyn Timberlake, Pietro Tiraboschi, Emily Todd, Philip Van Damme, Mathieu Vandenbulcke, Michele Veldsman, Ana Verdelho, Jorge Villanua, Jason Warren, Carlo Wilke, Ione Woollacott, Elisabeth Wlasich, Henrik Zetterberg, Miren Zulaica

**Affiliations:** 1 Division of Affinity Proteomics, Department of Protein Science, KTH Royal Institute of Technology, SciLifeLab, Stockholm, Sweden; 2 Swedish FTD Initiative, Stockholm, Sweden; 3 Department of Neurobiology, Care Sciences and Society, Division of Neurogeriatrics, Karolinska Institutet, Unit of Hereditary Dementias, Theme Aging, Karolinska University Hospital, Solna, Sweden; 4 Unit for Hereditary Dementias, Theme Aging, Karolinska University Hospital, Solna, Sweden; 5 Department of Neurology, Erasmus Medical Centre, Rotterdam, Netherlands; 6 Department of Neurodegenerative Diseases, Hertie-Institute for Clinical Brain Research and Center of Neurology, University of Tübingen, Tübingen, Germany; 7 Center for Neurodegenerative Diseases (DZNE), Tübingen, Germany; 8 Alzheimer’s disease and Other Cognitive Disorders Unit, Neurology Service, Hospital Clínic, Institut d’Investigacións Biomèdiques August Pi I Sunyer, University of Barcelona, Barcelona, Spain; 9 Cognitive Disorders Unit, Department of Neurology, Donostia University Hospital, San Sebastian, Gipuzkoa Spain; 10 Neuroscience Area, Biodonostia Health Research Institute, San Sebastian, Gipuzkoa Spain; 11 Department of Clinical Neurological Sciences, University of Western Ontario, London, Ontario Canada; 12 Sunnybrook Health Sciences Centre, Sunnybrook Research Institute, University of Toronto, Toronto, Canada; 13 Tanz Centre for Research in Neurodegenerative Diseases, University of Toronto, Toronto, Canada; 14 Laboratory for Cognitive Neurology, Department of Neurosciences, KU Leuven, Leuven, Belgium; 15 Neurology Service, University Hospitals Leuven, Leuven, Belgium; 16 Leuven Brain Institute, KU Leuven, Leuven, Belgium; 17 Clinique Interdisciplinaire de Mémoire, Département des Sciences Neurologiques, CHU de Québec, and Faculté de Médecine, Université Laval, QC, Canada; 18 Fondazione IRCCS Ospedale Policlinico, Milan, Italy; 19 University of Milan, Centro Dino Ferrari, Milan, Italy; 20 Centre for Neurodegenerative Disorders, Department of Clinical and Experimental Sciences, University of Brescia, Brescia, Italy; 21 Nuffield Department of Clinical Neurosciences, Medical Sciences Division, University of Oxford, Oxford, UK; 22 Department of Brain Sciences, Imperial College London, London, UK; 23 Division of Neuroscience and Experimental Psychology, Wolfson Molecular Imaging Centre, University of Manchester, Manchester, UK; 24 Departments of Geriatric Medicine and Nuclear Medicine, University of Duisburg- Essen, Duisburg, Germany; 25 Department of Psychiatry, Douglas Mental Health University Institute, McGill University, Montreal, Québec Canada; 26 McConnell Brain Imaging Centre, Montreal Neurological Institute, McGill University, Montreal, Québec Canada; 27 Department of Neurodegenerative Disease, Dementia Research Centre, UCL Institute of Neurology, Queen Square, London, UK

**Keywords:** Cerebrospinal fluid, Neurofilament medium polypeptide (NEFM), Neuronal pentraxin 2 (NPTX2), Neurosecretory protein VGF (VGF), Aquaporin 4 (AQP4), LASSO, Random forest, Suspension bead array

## Abstract

**Background:**

A detailed understanding of the pathological processes involved in genetic frontotemporal dementia is critical in order to provide the patients with an optimal future treatment. Protein levels in CSF have the potential to reflect different pathophysiological processes in the brain. We aimed to identify and evaluate panels of CSF proteins with potential to separate symptomatic individuals from individuals without clinical symptoms (unaffected), as well as presymptomatic individuals from mutation non-carriers.

**Methods:**

A multiplexed antibody-based suspension bead array was used to analyse levels of 111 proteins in CSF samples from 221 individuals from families with genetic frontotemporal dementia. The data was explored using LASSO and Random forest.

**Results:**

When comparing affected individuals with unaffected individuals, 14 proteins were identified as potentially important for the separation. Among these, four were identified as most important, namely neurofilament medium polypeptide (NEFM), neuronal pentraxin 2 (NPTX2), neurosecretory protein VGF (VGF) and aquaporin 4 (AQP4). The combined profile of these four proteins successfully separated the two groups, with higher levels of NEFM and AQP4 and lower levels of NPTX2 in affected compared to unaffected individuals. VGF contributed to the models, but the levels were not significantly lower in affected individuals. Next, when comparing presymptomatic *GRN* and *C9orf72* mutation carriers in proximity to symptom onset with mutation non-carriers, six proteins were identified with a potential to contribute to a separation, including progranulin (GRN).

**Conclusion:**

In conclusion, we have identified several proteins with the combined potential to separate affected individuals from unaffected individuals, as well as proteins with potential to contribute to the separation between presymptomatic individuals and mutation non-carriers. Further studies are needed to continue the investigation of these proteins and their potential association to the pathophysiological mechanisms in genetic FTD.

**Supplementary Information:**

The online version contains supplementary material available at 10.1186/s13024-021-00499-4.

## Background

Frontotemporal dementia (FTD) is a group of neurodegenerative diseases that typically displays a younger age at onset compared to other dementias [1]. A correct diagnosis is crucial to begin future disease-modifying treatments in the early phases of the disease. Protein biomarkers have the potential to aid the clinical assessment of patients and increase the knowledge about the molecular changes preceding symptom onset [2]. For this purpose, it is useful to study the genetic forms of FTD, including presymptomatic mutation carriers, to uncover early disease processes.

Different proteins in cerebrospinal fluid (CSF) and plasma have been suggested as potential biomarkers for FTD [3]. One of the most studied fluid biomarkers for symptom onset in FTD is neurofilament light chain, (NEFL, also known as NfL) [4]. Elevated CSF levels of NEFL is a marker of neuroaxonal damage [5] and has been investigated in a wide range of neurological conditions [6, 7]. Other biomarkers for FTD require further evaluation in additional cohorts to assess their potential [8–13].

A challenge in FTD research is the clinical, genetic and neuropathological diversity among patients which sometimes overlap with other neurodegenerative diseases. The different phenotypes (behavioural variant, primary progressive aphasias etc.), monogenic causes and aggregated proteins in brain tissue (mainly TAR DNA-binding protein 43; TDP-43 or tau) can challenge the ambitions to find fluid biomarkers for FTD. By utilizing machine learning based algorithms, patterns can be recognized which can be used to identify a panel of proteins that together have the potential to distinguish between different subgroups of FTD. Such a panel of proteins, instead of a single biomarker, will likely better resemble the complex and multifactorial nature of FTD.

In this study, we have used an antibody-based proteomics approach to measure proteins in CSF in a large, well-described cohort from the GENetic Frontotemporal dementia Initiative (GENFI) study [14]. Our aims were to identify panels of proteins and evaluate their potential to distinguish (I) affected individuals from unaffected individuals, and (II) presymptomatic mutation carriers from mutation non-carriers. In addition, identifying proteins altered in mutation carriers can bring further insight into important pathophysiological mechanisms in the disease development of genetic FTD.

## Methods

### Sample cohort

The sample cohort consisted of 221 participants recruited as a part of the GENFI study [14]. Baseline cerebrospinal fluid (CSF) samples were collected at 15 centres from year 2012 to 2019 according to a GENFI standardized protocol. Participants were enrolled in GENFI because they had a 50% risk of FTD due to a pathogenic mutation in a first degree relative in one of the following genes: chromosome 9 open reading frame 72 (*C9orf72*), progranulin (*GRN*), microtubule associated protein tau (*MAPT*) or TANK-binding kinase 1 (*TBK1*). Among the 221 participants, 47 were symptomatic individuals which hereafter will be annotated as affected mutation carriers (AMC), 98 were presymptomatic mutation carriers (PMC) and 76 were mutation non-carriers (NC). The PMC and NC will together be annotated as unaffected individuals. The clinical phenotype of AMC included behavioural variant FTD (bvFTD, *n* = 32) [15], primary progressive aphasia (PPA, *n* = 7) [16] and amyotrophic lateral sclerosis (ALS, *n* = 5) [17] (four with ALS and one with FTD-ALS). The remaining three affected participants included two with the phenotype progressive supranuclear palsy (PSP) and one with dementia that was not otherwise specified (D-NOS). When comparing CSF protein levels between affected (AMC) and unaffected individuals (PMC + NC), only AMC with bvFTD or PPA were included in the analysis to reduce phenotypic heterogeneity.

The age at onset ranged from 35 to 73 years, with a mean age of 58 years. The age and sex distribution for AMC, PMC and NC are presented in Table 1**,** as well as the mutated genes for the mutation carrier groups. For AMC, age at onset and clinical phenotype are also presented. No significant differences were observed for the sex distribution (Fisher’s exact test). One-way ANOVA was used to assess differences in age, which was observed between affected and unaffected participants, with older individuals in AMC compared to both PMC and NC (Tukey’s Honestly Significant Difference post hoc test). There was no statistically significant difference in age between PMC and NC. Years to expected onset was calculated based on the mean age at disease onset in the respective genetic groups (*C9orf72*, *GRN*, *MAPT*) according to Moore et al. 2020 [18].

**Table 1:** Demographic and clinical data

		Total	Affected mutation carriers	Presymptomatic mutation carriers	Non-carriers	*p*-value
Number of individuals		221	47	98	76	–
Sex distribution [F/M]		119/102	20/27	58/40	41/35	0.17^a^
Age [mean ± SD (range)]		50 ± 14 (20–76)	62 ± 9 (38–76)	46 ± 12 (20–74)	47 ± 13 (20–69)	3e-14^b^
Age at onset [mean ± SD (range)]			58 ± 9 (35–73)			–
Mutation [N (%)]	*C9orf72*		27 (57)	41 (42)		
	*GRN*		12 (26)	38 (39)		
	*MAPT*		7 (15)	16 (16)		
	*TBK1*		1 (2)	3 (3)		
Clinical phenotype [N (%)]	bvFTD		32 (68)			
	PPA		7 (15)			
	Other^c^		8 (17)			

Age measured in years

*AMC* affected mutation carriers, *PMC* presymptomatic mutation carriers, *NC* non-carriers, *C9orf72* chromosome 9 open reading frame 72, *GRN* progranulin, *MAPT* microtubule associated protein tau, *TBK1* TANK-binding kinase 1, *bvFTD* behavioural variant frontotemporal dementia, *PPA* primary progressive aphasia, *FTD-ALS* frontotemporal dementia with amyotrophic lateral sclerosis, *PSP* progressive supranuclear palsy, *D-NOS* dementia not otherwise specified

^a^Fisher’s exact test

^b^ANOVA. Tukey’s Honestly Significant Difference (Tukey’s HSD) post-hoc test was performed for pairwise comparisons (AMC vs NC, *p* = 3e-11; AMC vs PMC, *p* = 2e-13; PMC vs NC, *p* = 0.8)

^c^Other clinical phenotypes included ALS (*n* = 4), FTD-ALS (*n* = 1), PSP (*n* = 2), D-NOS (*n* = 1)

### Suspension bead array assay

CSF samples were collected by lumbar puncture into polypropylene tubes. Immediately after collection, the fluid was centrifuged at 963 xg for 10 min at 4 °C. The supernatant was aliquoted into polypropylene cryotubes and stored at − 80 °C. At the time for analysis, the CSF samples were labelled with a tenfold molar excess of biotin (NHS-PEG4-biotin, 21,329, Thermo Scientific) as previously described [19, 20]. The samples were distributed in 96-well PCR plates in a constrained randomized fashion, based on diagnostic group (AMC, PMC or NC), sex and age. Target proteins (*n* = 174) were selected based on previous internal published and unpublished neuroproteomic efforts [9, 21–26], complemented with additions from literature. Antibodies towards 169 of the 174 proteins were selected from the Human Protein Atlas project (www.proteinatlas.org) and antibodies towards the remaining five proteins were included from other providers. The majority of the antibodies were polyclonal rabbit antibodies, but three were monoclonal mouse antibodies and two were polyclonal goat antibodies. The antibodies were conjugated onto carboxylated color-coded magnetic beads (Magplex, Luminex corporation) using NHS-EDC chemistry, as described previously [20, 27, 28], where each bead identity corresponds to one antibody. The antibody coupled beads were subsequently pooled to form the suspension bead array. Next, the biotinylated CSF samples were further diluted, and heat treated at 56 °C for 30 min before they were combined with the suspension bead array in a 384-well plate and incubated overnight. Detection was enabled by the addition of a streptavidin conjugated fluorophore (Streptavidin R-Phycoerythrin Conjugate, Invitrogen), and the readout was performed in a Flexmap 3D instrument (Luminex corporation). After a stringent quality control (inter-assay correlation and correlation to negative control), we selected 111 proteins for further statistical analysis (Supplementary Table 1).

### Data analysis

The open source software R (version 4.0) [29] was used for data processing, analysis and illustrations. The analysis focused on two main comparisons: affected vs unaffected individuals and PMC vs NC. The following packages were used, in additions to the specific functions listed below: stats, tidyverse [30], ggbeeswarm, and ggpubr.

#### Quality control and normalization

The data was normalised to diminish the effects of time delay during readout. A robust linear regression (*rlm, MASS* [31]) was performed, and the obtained residuals were added to the median signal intensity per protein. In addition, a second normalisation step was performed to reduce potential differences between plates [32].

#### Predictive model building and feature selection

The Least absolute shrinkage and selection operator (LASSO) [33] and Random forest models were used for feature selection for downstream analyses. LASSO models (*cv.glmnet, glmnet* [34]) were constructed based on a training set consisting of 2/3 of the samples. The tuning parameter lambda was optimised to give the minimum cross-validation error. The performance of the model was assessed using a test set (the remaining 1/3 of the samples) and the area under the curve (AUC) of receiver operating characteristic analyses (*roc, pROC* [35]) with the optimal cut-off determined using Youden’s index [36] (*coords, pROC* [35]). The stability of the LASSO models was assessed by creating 1000 models using the same training set but different seeds during the cross-validation. In addition, Random forest models [37] (*randomForest, randomForest* [38]) were constructed based on the full data set. The number of trees was selected to minimise the out-of-bag (OOB) error rate and the number of random variables used in each tree was optimised using the tuning function (*tuneRF, randomForest* [38]). The performance of the model was assessed using AUC for the OOB samples. The mean decrease accuracy (mda), i.e. the difference in accuracy between a model with the actual values for a protein and a model where the data has been randomly shuffled, was used as an indicator of the importance of each protein. The stability of the Random forest models was assessed by creating 1000 models with different seeds, but the same selected number of trees and number of random variables. The mean mda (of 1000 models) was calculated per protein. We aimed for a relatively small number of proteins to be included in the panels to enable robust profiles and arbitrarily cut-offs based on this premise were therefore selected. When comparing affected and unaffected individuals, we focused on proteins present in at least 20% of the models from LASSO, or with a mean mda above 8 from Random forest. For the comparison of PMC and NC, we focused on a smaller list of proteins that were present in at least 80% of the models from LASSO or with a mean mda above 5 from Random forest.

#### Statistics and visualizations

The protein panels selected by LASSO and Random forest were further investigated in several ways. The potential univariate differences of protein levels between the different sample groups (affected vs unaffected and PMC vs NC) were evaluated using Wilcoxon rank sum test and the correlation between different protein levels was calculated using non-parametric Spearman’s correlation coefficients. *P*-values below 0.05 were considered significant. Principal component analysis (PCA) was performed on log-transformed data (*PCA, FactoMineR* [39]; *fviz_pca_ind, factoextra* [40]). Hierarchical clustering of PC1 and PC2 was performed using Euclidean distances and average linkage.

## Results

### Exploration of a panel of proteins to identify symptomatic FTD

LASSO and Random forest were used to identify proteins with the combined ability to distinguish affected from unaffected individuals. The affected individuals included FTD patients expressing either bvFTD or PPA, and the unaffected individuals included both PMC and NC.

#### Protein selection based on LASSO models

The training data used for constructing a LASSO model included 26 affected individuals and 117 unaffected individuals (2/3 of the samples). A five-fold cross validation of the training data was performed to optimize the tuning parameter lambda in order to obtain the optimal trade-off between bias and variance. Three proteins, neurofilament medium polypeptide (NEFM, also known as NfM), neuronal pentraxin 2 (NPTX2) and apolipoprotein E isoform 4 (APOE4), were selected by LASSO when constructing the model. Next, the prediction performance of the model was assessed using the test data which included 13 affected and 57 unaffected individuals (1/3 of the samples). A ROC curve was obtained with an AUC of 0.90. The model correctly predicted the diagnostic status of 67/70 samples in the test set, with two false negatives and one false positive sample (i.e. two affected were classified as unaffected and one unaffected as affected).

Next, the stability of the LASSO model was assessed by creating 1000 models based on the training data. The AUC obtained when predicting the test data ranged from 0.89 to 0.98. The seven proteins with highest score from LASSO were NEFM (100%), NPTX2 (71.6%), APOE4 (55.6%), neurosecretory protein VGF (VGF, 40.2%), translocation protein SEC63 homolog (SEC63, 40.2%), apolipoprotein A1 (APOA1, 31.5%) and aquaporin 4 (AQP4, 26.5%) (Table 2). The models with the lowest AUC (0.89) correspond to the models where only NEFM was included.

**Table 2:** Proteins selected from LASSO or random forest when comparing affected and unaffected individuals

Protein	Description	Uniprot	Antibody	LASSO % selected	Random forest Mean mda
NEFM*	Neurofilament medium polypeptide	P07197	HPA022845	100	28.1
NPTX2*	Neuronal pentraxin 2	P47972	HPA049799	71.6	8.4
VGF*	Neurosecretory protein VGF	O15240	HPA055177	40.2	9.2
AQP4*	Aquaporin 4	P55087	HPA014784	26.5	11.3
APOE4	Apolipoprotein E isoform 4	Q8TCZ8	M067–3	55.6	0.8
SEC63	Translocation protein SEC63 homolog	Q9UGP8	HPA053295	40.2	3.1
APOA1	Apolipoprotein A1	P02647	HPA046715	31.5	4.2
PTPRN2	Protein tyrosine phosphatase, receptor type N2	Q92932	HPA007255	8.8	8.2
CTSS	Cathepsin S	P25774	HPA002988	7.6	9.4
SERPINA3	Serpin family A member 3	P01011	HPA000893	0	12.8
C4A/B	Complement C4A, complement C4B	P0C0L4, P0C0L5	HPA046356	0	11.0
AMPH	Amphiphysin	P49418	HPA019829	0	9.5
SPP1	Secreted phosphoprotein 1	P10451	NBP2–37423	0	8.8
CD14	Monocyte differentiation antigen CD14	P08571	HPA002035	0	8.5

Asterisk next to the protein name show which proteins were selected by both LASSO and Random forest. Proteins with grey numbers in the LASSO or Random forest columns did not meet the selected cut-off for that model. LASSO indicates in how many models a protein was selected (% out of 1000 models). Random forest indicates the mean mda from 1000 models

#### Protein selection based on random forest models

In addition to the LASSO models, Random forest was utilised to select a panel of proteins with the potential to distinguish affected from unaffected individuals. First, one Random forest model was constructed and its prediction ability using the OOB samples was subsequently evaluated. The AUC of the model was 0.94 and it correctly classified 194 out of the 213 included samples (34/39 affected individuals, 160/174 unaffected individuals).

Furthermore, the stability of the Random forest models was assessed by creating 1000 forests. AUC varied from 0.93 to 0.95. Eleven proteins were defined by Random forest as the most contributing factors (Table 2) and were selected for further analysis.

#### Four proteins selected by both LASSO and random forest

**Fig. 1:**
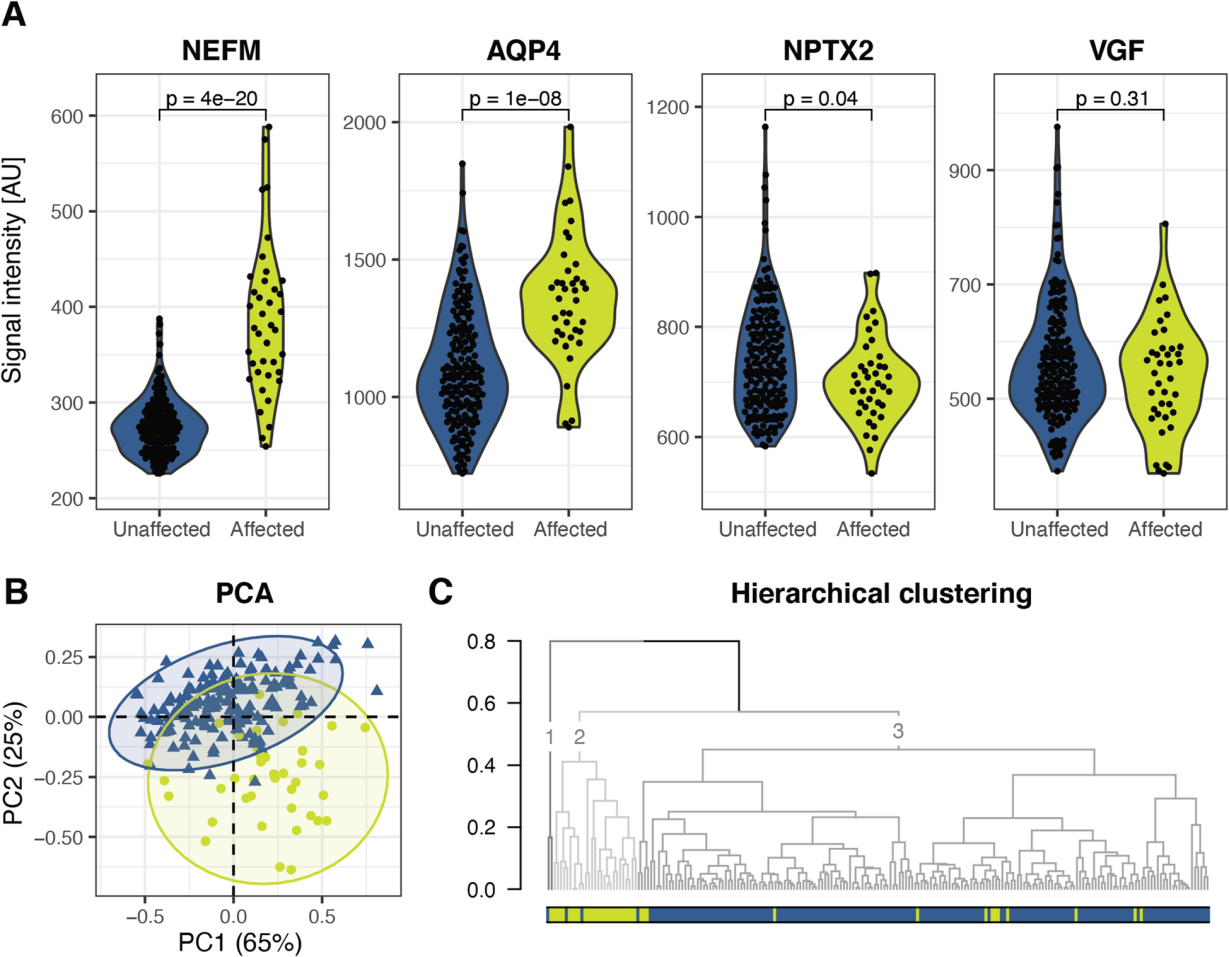
Affected vs unaffected. Four proteins (NEFM, AQP4, NPTX2 and VGF) selected by both Random forest and LASSO when comparing affected and unaffected individuals. Yellow and circles = affected individuals (*n* = 39), blue and triangles = unaffected individuals (*n* = 174). **A** Violin plots for NEFM, AQP4, NPRX2 and VGF, with *p*-values from Wilcoxon rank sum test. **B** A PCA plot based on the four selected proteins. **C** A hierarchical clustering based on PC1 and PC2. NEFM, neurofilament medium polypeptide; AQP4, apolipoprotein E isoform 4; NPTX2, neuronal pentraxin 2; VGF, neurosecretory protein VGF; AU, arbitrary units; PCA, principal component analysis

A total of 14 proteins were selected by either LASSO or Random forest (Table 2), and four of these proteins were selected by both models, NEFM, NPTX2, VGF and AQP4. The differences in levels between the affected and unaffected individuals for these four proteins are visualised in Fig. 1A and the remaining ten proteins in Supplementary Fig. 1. The protein levels of NEFM and AQP4 were significantly higher in the affected group, while NPTX2 was significantly lower, compared to the unaffected individuals. The mean VGF levels in the affected group was lower than the mean in the unaffected group, although it did not reach statistical significance. A principal component analysis of these four proteins revealed that 90% of the distribution of differences could be explained by principal component 1 and 2 (Fig. 1B). Affected individuals mainly cluster in the bottom right part of the plot while unaffected individuals cluster in the top left corner. Furthermore, the four proteins’ combined potential to distinguish the affected individuals from the unaffected individual was evaluated using a hierarchical clustering approach (Fig. 1C). When dividing the samples into three clusters, we identified one very small cluster (1) with only two individuals (one affected and one unaffected individual), one cluster (2) with 93% affected individuals (affected *n* = 25, unaffected *n* = 2) and one cluster (3) with 93% unaffected individuals (affected *n* = 13, unaffected *n* = 171). The group of unaffected individuals was further investigated by observing protein levels and cluster distribution of PMC and NC separately (Supplementary Fig. 2). There were no differences in protein levels between PMC and NC for NEFM, NPTX2, VGF or AQP4, nor did they separate in the PCA analysis. PMC and NC cluster together in cluster 3 without any apparent pattern. The two unaffected individuals clustering together with the AMC (cluster 2) are both NC, and one PMC is clustering together with one AMC in cluster 1. Next, the distribution of the genetic causes and clinical phenotypes of the affected individuals were investigated, but no pattern could be identified that indicated a large difference between the genetic groups or between bvFTD and PPA connected to the levels of NEFM, NPTX2, VGF and AQP4 (Supplementary Fig. 2B). Furthermore, a PCA analysis based on these four proteins was performed, where all phenotypes were included, i.e. the affected individuals with the clinical phenotypes bvFTD and PPA as well as ALS, FTD-ALS, PSP or D-NOS (Supplementary Fig. 3). All AMC cluster together, regardless of clinical phenotype.

A heatmap (Supplementary Fig. 4) of the spearman correlations between the 14 proteins presented in Table 2 demonstrated a strong correlation between NPTX2, VGF and PTPRN2 (NPTX2-VGF rho = 0.83, NPTX2-PTPRN2 rho = 0.79, VGF-PTPRN2 rho =0.89). A large cluster with moderate correlations included AQP4, AMPH, CD14, C4A/B, NEFM, SERPINA3, CTSS, SPP1, where AQP4 and AMPH had the strongest individual correlation (rho = 0.86). SERPINA3 had a strong correlation to APOA1 (rho = 0.81), CD14 (rho = 0.74) and C4A/B (rho = 0.72). APOE4 had a weak correlation (rho < 0.40) with all other proteins.

### CSF profiles in presymptomatic mutation carriers versus mutation non-carriers

**Fig. 2:**
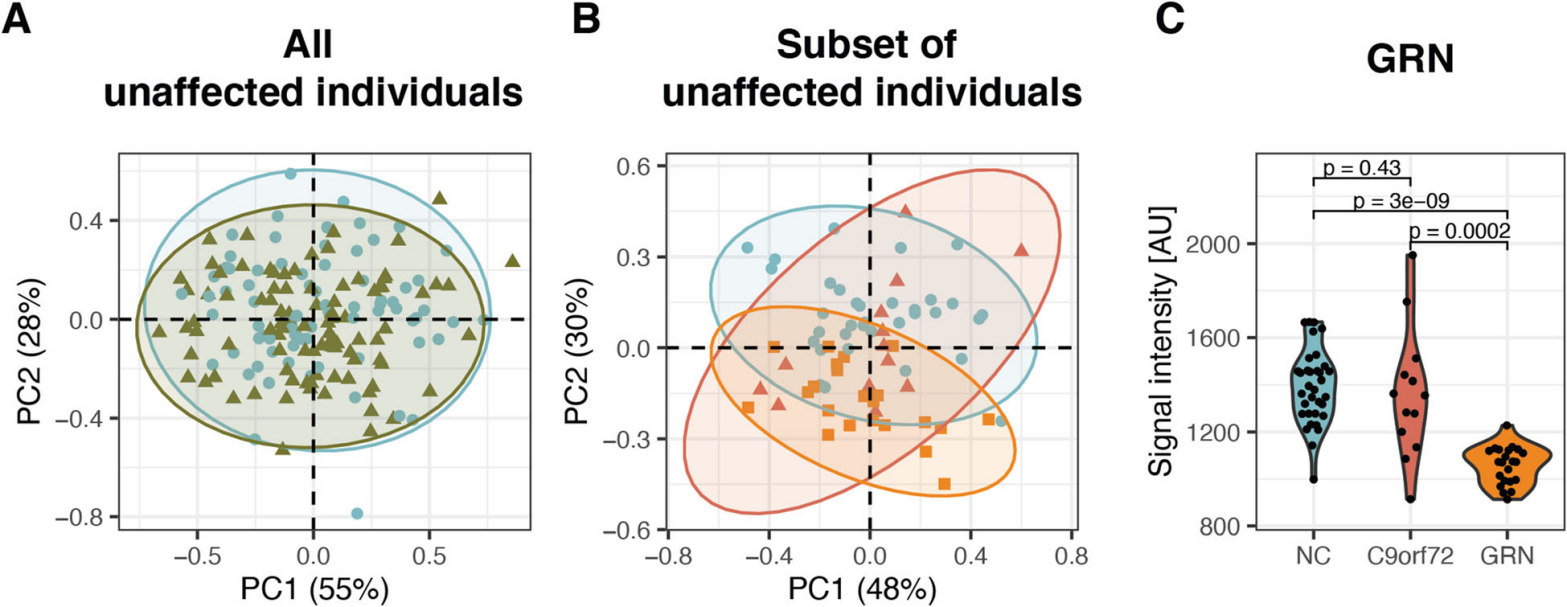
PMC vs NC. **A** PCA based on four proteins (GRN, KNG1, AQP4 and UPF0606 protein KIAA1549L) selected by either LASSO or Random forest when all PMC (*n* = 98, green triangles) and NC (*n* = 76, blue dots) were included. **B** PCA based on six proteins (GRN, TARDBP, KNG1, HBEGF, MBP and CLSTN1) selected by either LASSO or Random forest when the selection was based on age and pathology filtered individuals (NC *n* = 34 blue dots, PMC C9orf72 *n* = 13 red triangles, PMC GRN *n* = 22 orange squares). **C** One protein was selected by both LASSO and Random forest: GRN, presented with *p*-values from Wilcoxon rank sum test. Same number of individuals included as in B. NC, non-carriers; C9orf72, chromosome 9 open reading frame 72; GRN, progranulin; AU, arbitrary units

The construction of LASSO and Random forest models for the comparison of PMC vs NC was performed in the same way as described in previous sections (affected individuals vs unaffected individuals). However, when comparing PMC and NC, only one protein, progranulin (GRN) was selected by both LASSO and Random forest. In addition to GRN, one protein (kininogen 1, KNG1) was selected from the LASSO models and two proteins (AQP4 and UPF0606 protein KIAA1549L) from the Random forest analysis. A PCA plot based on the four proteins are shown in Fig. 2A. However, no separation between the PMC and NC could be observed (nor between any of the genetic groups, Supplementary Fig. 5).

To optimise the analysis, only PMC with expected present or future TDP-43 pathology and less than 10 years to expected symptom onset (based on the mean age at disease onset in the respective genetic groups according to Moore et al. 2020 [18]) were selected (i.e. *C9orf72* mutation carriers older than 48 years and *GRN* mutation carriers older than 51 years). *TBK1* presymptomatic mutation carriers were excluded from this analysis as there were only three individuals and we currently lack good estimations of mean age at symptom onset for this group. In addition, NC older than 48 years were selected to match the ages of PMC. Thus, 13 PMC *C9orf72*, 22 PMC *GRN* and 34 NC were included in the models hereafter. The LASSO training data set included 43 individuals (20 PMC and 23 NC) and the test data set included 26 individuals (15 PMC and 11 NC). When the stability of the LASSO was assessed, the AUC obtained varied from 0.73 to 0.82. The four proteins with highest score from the LASSO models were GRN, TAR DNA binding protein 43 (TARDBP), KNG1 and heparin binding EGF like growth factor (HBEGF) (Table 3). In the Random forest models, the AUC ranged between 0.74 and 0.80 and the three proteins with highest score were GRN, myelin basic protein (MBP) and calsyntenin-1 (CLSTN1) (Table 3).

**Table 3:** Proteins selected when comparing age and pathology filtered PMC to NC

Protein	Description	Uniprot	Antibody	LASSO % selected	Random forest Mean mda
GRN*	Progranulin	P28799	AF2420	99.9	23.4
TARDBP	TAR DNA binding protein 43	Q13148	HPA070770	85.8	−0.1
KNG1	Kininogen 1	P01042	HPA001645	82.2	1.6
HBEGF	Heparin binding EGF like growth factor	Q99075	HPA053243	82.2	1.5
MBP	Myelin basic protein	P02686	HPA049222	0	5.0
CLSTN1	Calsyntenin-1	O94985	HPA012749	0	5.0

Selection from LASSO and Random forest when comparing PMC (*n* = 35) and NC (*n* = 34) (age and pathology filtered). Only GRN was selected by both LASSO and Random forest. Proteins with grey numbers in LASSO or Random forest columns did not meet the cut-off for that model. LASSO indicates in how many models a protein was selected (% out of 1000 models). Random forest indicates the mean mda from 1000 models

#### Only GRN selected by both LASSO and random forest

A total of six proteins were selected by either LASSO or Random forest (Table 3). A PCA for the combination of these six proteins are shown in Fig. 2B. The protein with the highest contribution to PC1 was KNG1 (40%), and the main contributing protein to PC2 was GRN (41%). Similar to the model with all PMC and NC, only GRN was selected by both models. The differences in GRN levels between NC, PMC *C9orf72* and *GRN* mutation carriers are shown in Fig. 2C, and the protein levels of the five proteins selected by LASSO or Random forest are shown in Supplementary Fig. 6. As expected, the level of GRN was lower in *GRN* mutation carriers which could be separated from non-carriers in the PCA, but there was no difference between *C9orf72* mutation carriers and NC. The strongest correlation (Supplementary Fig. 7) between these six proteins listed in Table 3 was observed between MBP and TARDBP (rho = 0.82) and the protein with the strongest correlation with GRN was CLSTN1 (rho = 0.61).

## Discussion

This study aimed to identify panels of CSF proteins with the potential to distinguish patients with FTD from unaffected individuals, as well as PMC from NC, using the multivariate statistical methods LASSO and Random forest. The protein levels were measured in CSF from 221 individuals by utilizing an antibody-based suspension bead array. Four proteins were identified as most important when comparing affected to unaffected individuals: NEFM, NPTX2, VGF and AQP4, and their combined profile successfully separated the two groups.

NEFM is one of the subunits in neurofilaments, together with NEFL and neurofilament heavy polypeptide (NEFH, also known as NfH). NEFL is a general marker of neuroaxonal damage but with especially high levels in FTD and ALS [6] and is one of the most promising fluid biomarker of symptom onset and disease severity in FTD. The less studied larger subunits of neurofilament, NEFM and NEFH are also elevated in CSF in several neurodegenerative disorders, including FTD [9, 13, 41]. The present study supports previous findings that the levels of NEFM in CSF is higher in patients with FTD than in controls and further studies will elucidate the potential value of measuring NEFM in addition to NEFL.

Also, NPTX2 and VGF have previously been identified as potential FTD biomarkers in CSF [8–10, 13]. NPTX2, together with neuronal pentraxin 1 (NPTX1) and neuronal pentraxin receptor (NPTXR), are important for synaptic homeostasis and plasticity [42] and synaptic dysfunction is a suggested pathological mechanism for FTD. CSF levels of NPTX2 have been shown to negatively correlate with disease severity and grey matter volume in genetic FTD [8]. A reduction of NPTX2 in CSF is also observed in Alzheimer disease (AD) and already in individuals with mild cognitive impairment compared to controls [43–46]. Xiao et al. 2017 found an inverse correlation between NPTX2 levels in CSF and cognitive performance [43] and Soldan et al. 2019 identified a potential association between CSF NPTX2 and the salience attention network [44]. Similar to NPTX2, levels of VGF are also lower in CSF in FTD [9, 10] and AD [46–48]. Furthermore, CSF levels of both NPTX2 and VGF are lower in patients with dementia with Lewy bodies (DLB) compared to controls and patients with AD [49, 50]. VGF is related to synaptic function, and a strong correlation (rho = 0.70) between VGF and NPTX2 levels was previously observed in patients with DLB and AD by Boiten et al. 2020 [51], which is in concordance with the strong correlation (rho = 0.83) we observed in this FTD cohort. Further studies are needed to characterise the connection between VGF and NPTX2 and their role in neurodegenerative disorders.

The fourth and last protein being identified as highly important in the separation between affected and unaffected individuals was AQP4. AQP4 is the most abundant water channel in the brain and important for maintaining brain water homeostasis [52]. This bidirectional water channel is mostly localized in astrocytes close to blood vessels (perivascular) with a particularly high expression at the blood brain barrier and blood CSF barrier [53]. AQP4 has been suggested to play a role in several neurological diseases such as hydrocephalus, stroke and also AD either by an altered gene expression or a change in localisation [52].

When comparing affected and unaffected individuals we focused on affected individuals with bvFTD or PPA in order to reduce phenotypic heterogeneity. We did not observe a large difference between the two clinical phenotypes regarding the four proteins (NEFM, NPTX2, VGF and AQP4) selected based on their separation capacity between affected and unaffected individuals. Nor could we see any distinct deviation when the analysis was extended to the remaining affected individuals, i.e. the individuals with ALS, FTD-ALS, PSP or D-NOS. A more direct effort to find proteins separating different clinical phenotypes would be interesting but was not possible in this cohort due to the small number of patients expressing the respective clinical phenotypes.

The next step in our analysis was to compare PMC and NC, but no distinct differences between the groups were observed except for GRN. We decided to focus the analysis on PMC in proximity to their predicted symptom onset (< 10 years) and with an expected future or present TDP-43 pathology. Five proteins (TARDBP, KNG1, HBEGF, MBP and CLSTN1) were identified, in addition to GRN, that contributed to the separation between PMC and NC. However, additional studies are needed to further validate these protein profiles and elucidate their characteristics and potential association to pathophysiological mechanisms in genetic FTD.

Pathogenic variants of *GRN* cause haploinsufficiency which leads to a 50% reduction of GRN in mutation carriers (both presymptomatic and symptomatic) compared to non-carriers. This decrease in GRN levels is measurable in both plasma and CSF but the majority of studies of GRN levels has been performed in plasma partly due to the higher relative concentration observed in this less invasive sample material [3]. As expected, we observed significantly lower CSF levels of GRN in individuals with a pathogenic mutation in *GRN* compared to non-carriers.

TARDBP (also known as TDP-43) is the main component of the neuronal inclusions found at neuropathological examinations in the majority of FTD cases and almost all ALS cases [54]. It is an attractive protein to measure in CSF as it could have the possibility to distinguish FTD with TDP-43 pathology from FTD with tau-pathology in living patients since this can only be inferred in genetic and not sporadic cases. No validated method of detecting TDP-43 in CSF exists as of today. One recent study has detected pathological aggregates of TDP-43 in CSF of *C9orf72*, *GRN* and *TARDBP* mutation carriers but not in controls [55] which warrants further investigations into the potential use of TDP-43 as a biomarker for FTD with TDP-43 pathology. When comparing *GRN* and *C9orf72* PMC in proximity to symptom onset to NC, LASSO identified TARDBP as important and included the protein in 85.8% of the models. The only protein selected in more models was GRN. However, TARDBP was not regarded important in the Random forest models and we did not observe a significant difference in the univariate analysis.

The multiplexed single binder suspension bead array offers a high-throughput analysis. In order to fully confirm the target specificity there is a need for further validation and characterisation, utilizing more antibodies in either single binder assays or sandwich assay or other orthogonal methods such as parallel reaction monitoring (PRM) assays or epitope mapping. This is exemplified by the thorough characterisation of NEFM investigated using both sandwich assays [9] and PRM assay [23] and AQP4 with a sandwich assay [26]. All used antibodies from the Human Protein Atlas have been validated and confirmed to only bind its specific target in a protein array format (www.proteinatlas.org).

Univariate protein levels often overlap considerably between different sample groups while a panel of proteins have the potential to improve the discriminatory capacity between groups. We have used two machine learning based algorithms to identify such panels of proteins: LASSO and Random forest. The two methods have different intrinsic properties, for example how they handle correlating variables. Using both algorithms allow us to achieve more robust results since the four most important proteins were chosen in both models. LASSO performs regularisation in order to increase prediction accuracy. By removing less important features (variables, i.e. proteins), the interpretation of the model is improved. Random forest ensembles a large number of decision trees with randomly selected features and combines their predictions. The importance of each feature is indicated using the calculated mda per protein. One advantage with Random forest is that the accuracy of the model can be estimated from the OOB samples, i.e. samples in the original data but not included when building a particular tree. Both algorithms were able to successfully predict the classification of the samples when comparing affected and unaffected individuals. The AUC varied between 0.89 and 0.98 for 1000 LASSO models, and between 0.93 and 0.95 for 1000 Random forest models. In the prediction models for PMC and NC, the accuracy was lower (0.73–0.82). For some biomarkers, as for example GRN, the levels in CSF are most likely reduced already at birth due to haploinsufficiency. For others, such as dipeptide repeat proteins produced as a consequence of the *C9orf72* expansion, the temporal changes have not been ascertained and it is not clear when they deviate from normal levels in the asymptomatic stage. In this cohort, PMC were at different stages of their preclinical phase of FTD, with different number of years from estimated age at onset. The protein levels might vary greatly across individuals, which might influence the outcomes of our models. Furthermore, the sample size was reduced after filtering based on age and expected pathology. It is possible that we would reach a higher prediction accuracy between PMC and NC in a larger cohort, which would enable a better training set for the models. Moreover, it would be beneficial to use a longitudinal study design where samples from the same individuals are collected over many years. This would enable a more precise investigation of the temporal dynamics connected to FTD. Follow-up samples are being collected and will enable longitudinal analysis in the future. Since FTD is such a diverse disease, it would be beneficial to study distinct subgroups separately. It is not optimal to combine patients with different genetic causative pathways when trying to identify protein profiles with the potential to reflect pathophysiological processes in the brain. This requires large cohorts, and the small size of the subgroups (for example PMC with MAPT mutations) in this study limited such comparisons. However, the patterns observed for NEFM, AQP4, VGF and NPTX2 were further characterised based on genetic groups and no distinct clusters were observed based on the genetic cause. Future studies with even larger cohorts would of course, as always, be beneficial and could potentially enable a separation of the individuals into distinct genetic subgroups. Lastly, one advantage with research on genetic FTD is the ability to study PMC and the disease stages before symptom onset. However, the generalisability of the findings to sporadic FTD needs to be investigated in future studies. Furthermore, as both NEFM, NPTXR, VGF and AQP4 have been implicated in other neurodegenerative disease in addition to FTD, it cannot be stated here that the profiles of the proteins included in the panel are specific for FTD. Previous studies have investigated these four proteins separately and it remains to be explored if a combination of the four proteins has a higher discriminatory power between different diseases than each individual protein by themselves. Thus, the panel needs to be further investigated in other neurodegenerative cohorts.

## Conclusion

In conclusion, by using multivariate statistical methods to explore CSF levels of 111 proteins, we have identified a panel of four proteins (NEFM, AQP4, NPTX2 and VGF) which successfully distinguish most affected individuals from unaffected individuals. However, these four proteins were not able to separate between the different genes (mutation groups) or between the different clinical phenotypes. Furthermore, when focusing on PMC *GRN* and *C9orf72* close to expected symptom onset, we have identified five proteins (TARDBP, KNG1, HBEGF, MBP, CLSTN1) in addition to GRN, with the potential to contribute to the separation between PMC and NC. Continued exploration of these proteins, in independent cohorts, is needed in order to elucidate their potential association to FTD pathology.

## Supplementary Information

**Additional file 1.**

**Additional file 2.**

## Data Availability

The datasets used and/or analysed during the current study are available from the corresponding author on reasonable request.

## Abbreviations

AD: Alzheimer disease
ALS: Amyotrophic lateral sclerosis
AMC: Affected mutation carriers
AMPH: Amphiphysin
ANOVA: Analysis of variance
APOA1: Apolipoprotein A1
APOE4: Apolipoprotein E isoform 4
AQP4: Aquaporin 4
AUC: Area under curve
bvFTD: Behavioural variant FTD
C4A/B: Complement C4B, complement C4A
C9orf72: Chromosome 9 open reading frame 72
CD14: Monocyte differentiation antigen CD14
CLSTN1: Calsyntenin-1
CSF: Cerebrospinal fluid
CTSS: Cathepsin S
D-NOS: Dementia not otherwise specified
FTD: Frontotemporal dementia
GENFI: Genetic Frontotemporal dementia Initiative
GRN: Progranulin
HBEGF: Heparin binding EGF like growth factor
KNG1: Kininogen 1
LASSO: Least absolute shrinkage and selection operator
MAPT: Microtubule associated protein tau
MBP: Myelin basic protein
NC: Non-carriers
NEFH/NfH: Neurofilament heavy polypeptide
NEFL/NfL: Neurofilament light polypeptide
NEFM/NfM: Neurofilament medium polypeptide
NPTX2: Neuronal pentraxin 2
OOB: Out-of-bag
PC: Principal component
PCA: Principal component analysis
PMC: Presymptomatic mutation carriers
PPA: Primary progressive aphasia
PRM: Parallel reaction monitoring
PSP: Progressive supranuclear palsy
PTPRN2: Protein tyrosine phosphatase, receptor type N2
ROC: Receiver operating characteristic
SEC63: Translocation protein SEC63 homolog
SERPINA3: Serpin family A member 3
SPP1: Secreted phosphoprotein 1
TARDBP/TDP-43: TAR DNA-binding protein 43
TBK1: TANK-binding kinase 1
VGF: Neurosecretory protein VGF
