# Supplementary material for: A panel of CSF proteins separates genetic frontotemporal dementia from presymptomatic mutation carriers: a GENFI study
Source: Mol Neurodegener. 2021 Nov 27;16:79. doi: 10.1186/s13024-021-00499-4 (PMC8626910; doi:10.1186/s13024-021-00499-4)
Supplement: Supplementary file 2 — Additional file 2. [file 13024_2021_499_MOESM2_ESM.docx]

A panel of CSF proteins separates genetic frontotemporal dementia from presymptomatic mutation carriers: a GENFI study

# Supplementary figures


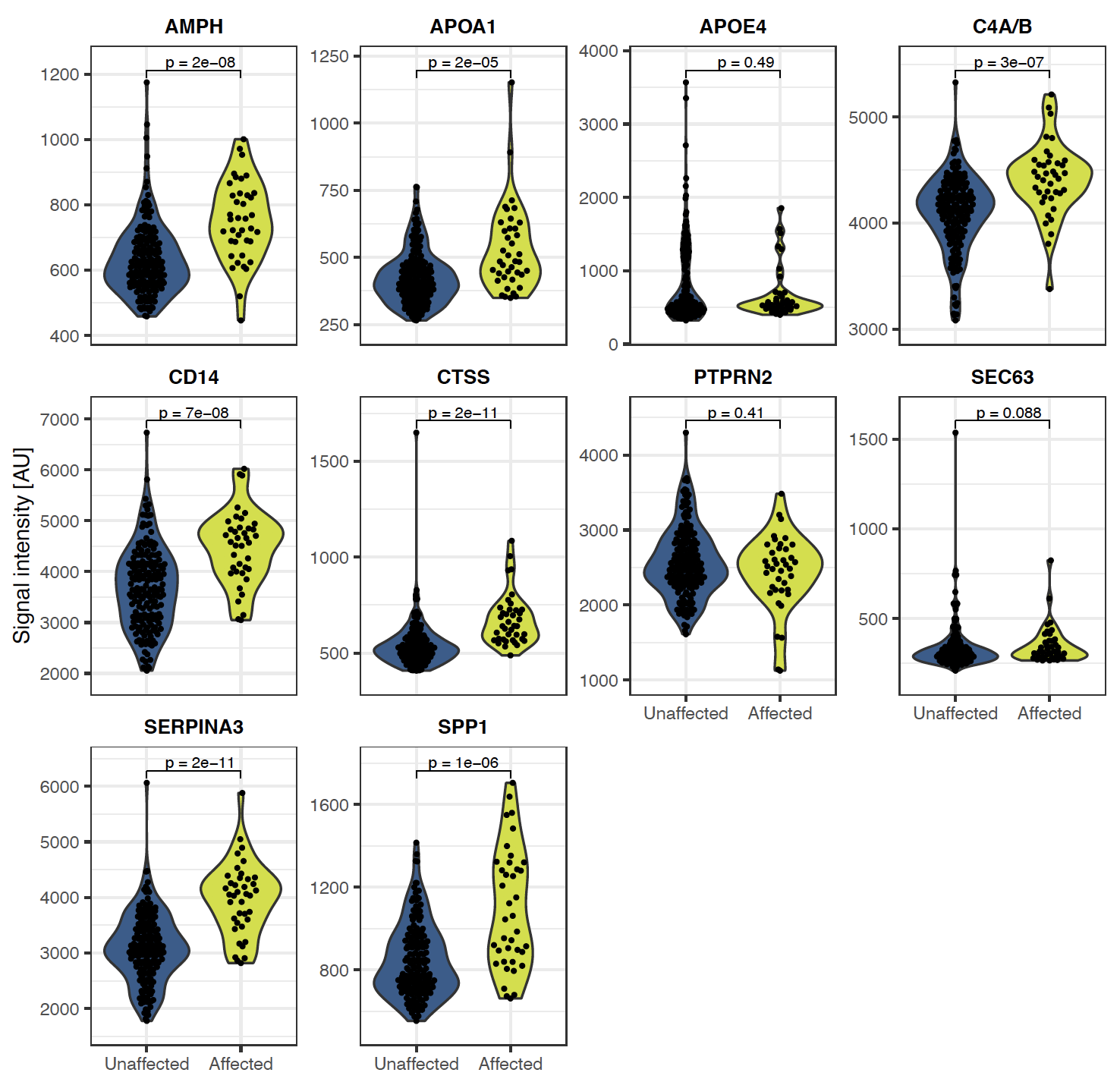


## *Supplementary Figure 1: Violin plots for the 10 proteins selected by LASSO or Random forest when comparing affected and unaffected individuals. The proteins selected by both LASSO and Random forest are visualized in Figure 1.*


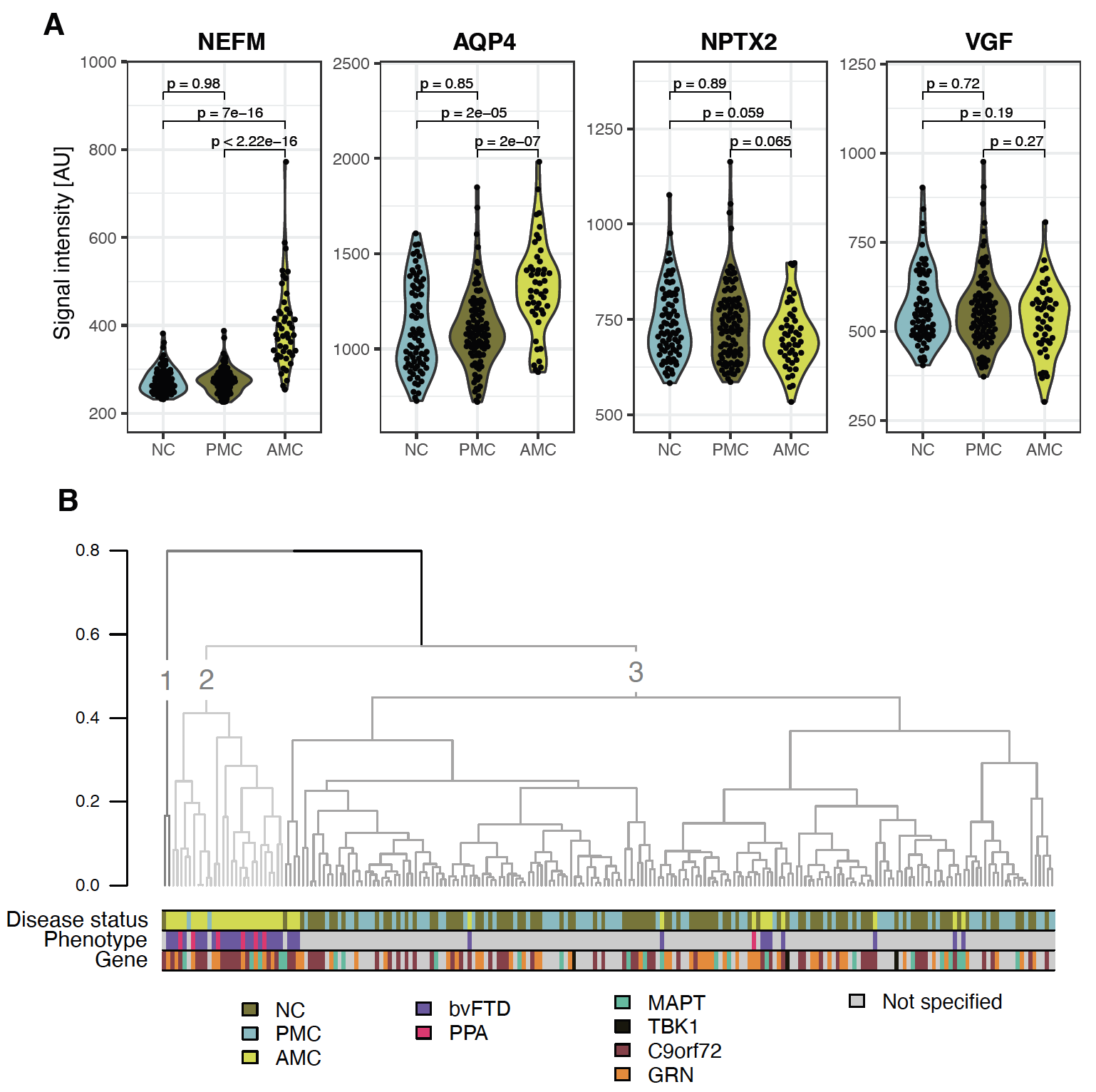


## *Supplementary Figure 2:*

Four proteins (NEFM, AQP4, NPTX2 and VGF) selected by both Random forest and LASSO when comparing affected and unaffected individuals. A) Violin plots for NEFM, AQP4, NPTX2 and VGF with p-values based on Wilcoxon rank sum tests. B) A hierarchical clustering based on PC1 and PC2 from PCA. One bar indicates the disease status (AMC, PMC or NC), one bar indicates the clinical phenotype for the AMC, and a third bar that indicates which mutation group each individual belongs to.


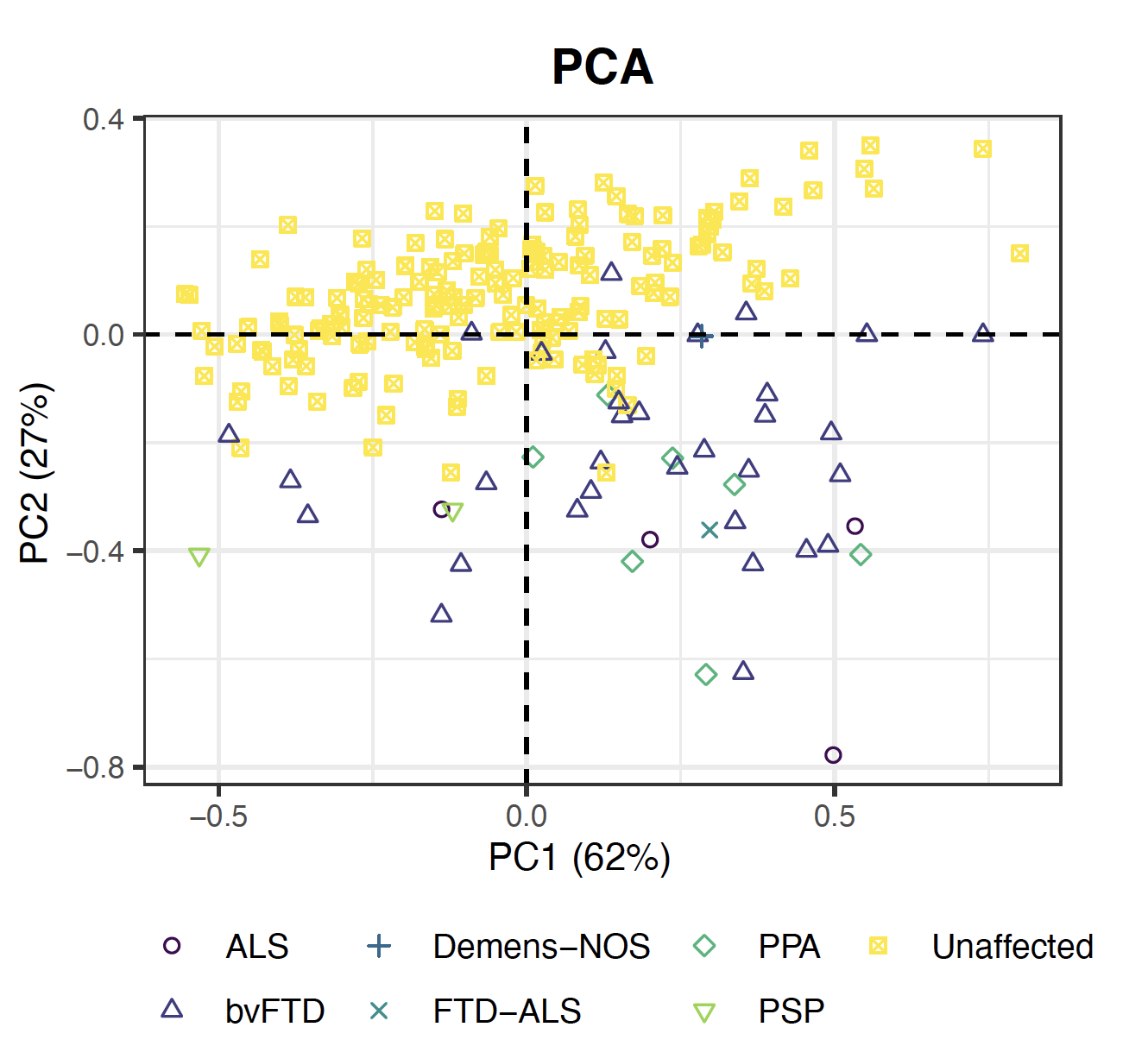


## *Supplementary Figure 3:*

*A PCA plot based on NEFM, NPTX2, VGF and AQP4 including all AMC, regardless of clinical phenotype.*


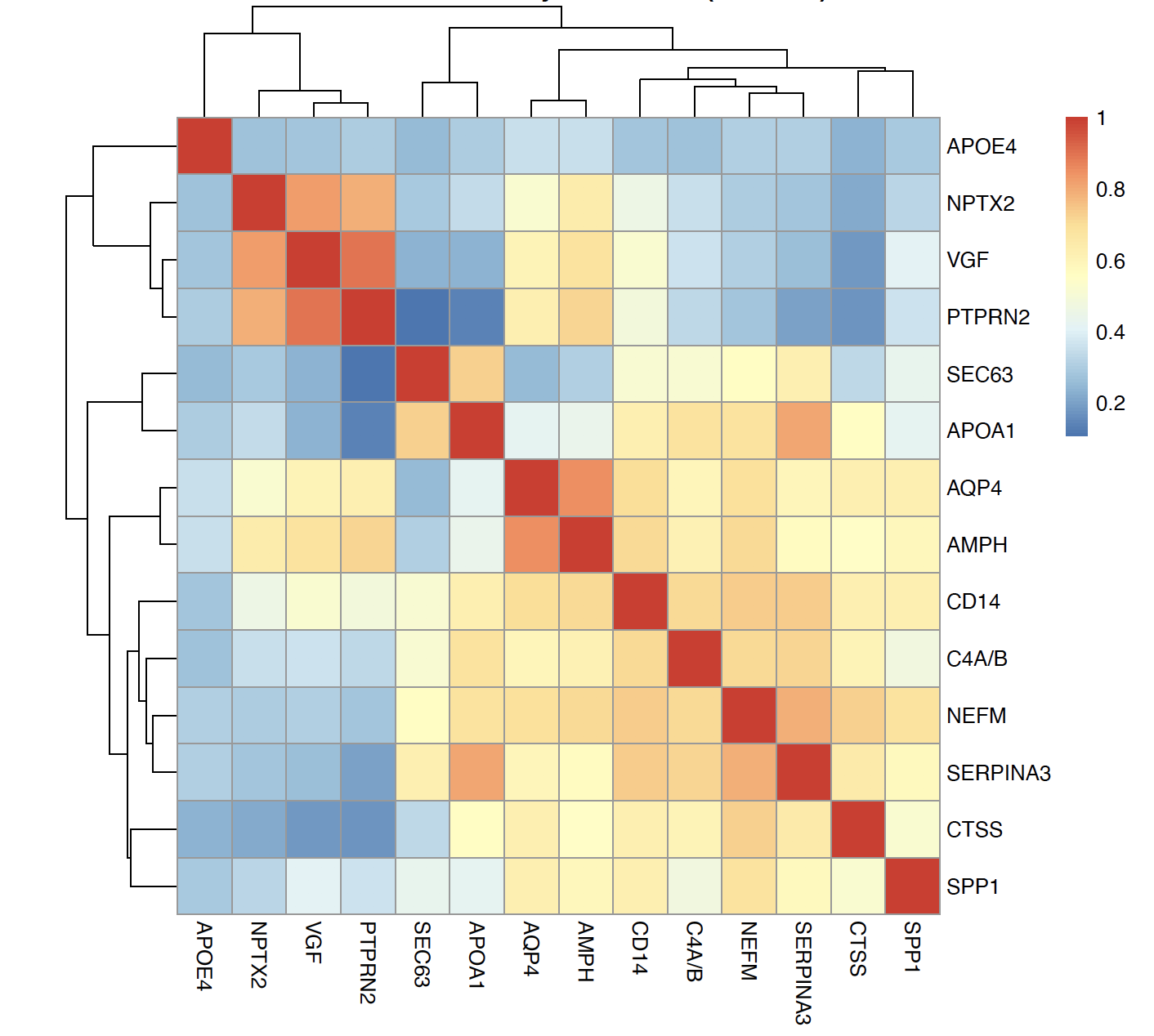


## *Supplementary Figure 4: The spearman correlation between the 14 proteins selected by either LASSO or Random forest when comparing affected individuals and unaffected individuals.*


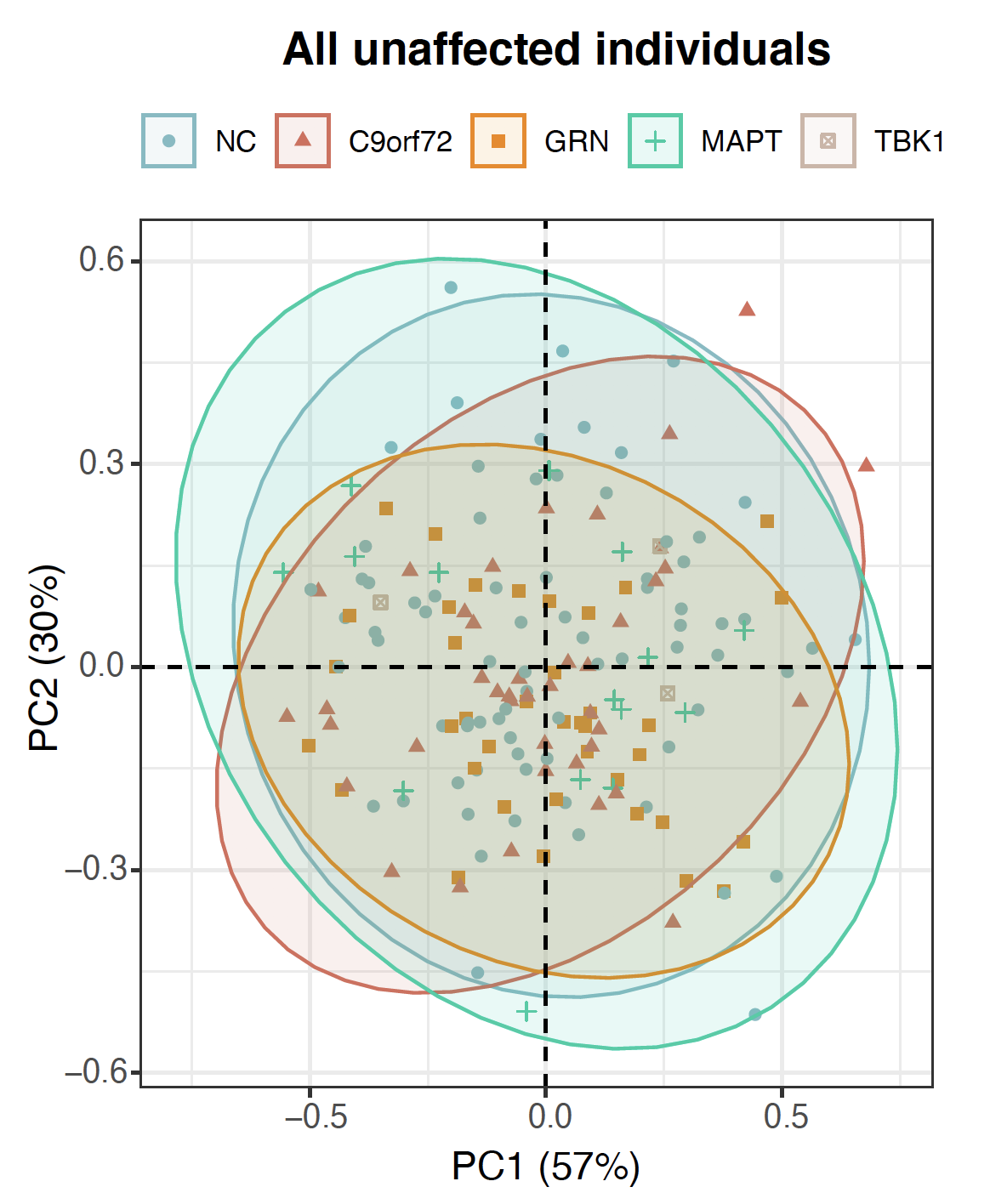


## *Supplementary Figure 5: A PCA plot of all unaffected individuals based on four proteins (GRN, KNG1, AQP4 and UPF0606 protein KIAA1549L) selected by either LASSO or Random forest. NC (n = 76, blue dots), PMC C9orf72 (n = 41, red triangles), PMC GRN (n = 38, orange squares), PMC MAPT (n = 16, green cross), PMC TBK1 (n = 3, brown squares with cross) were included.*

*
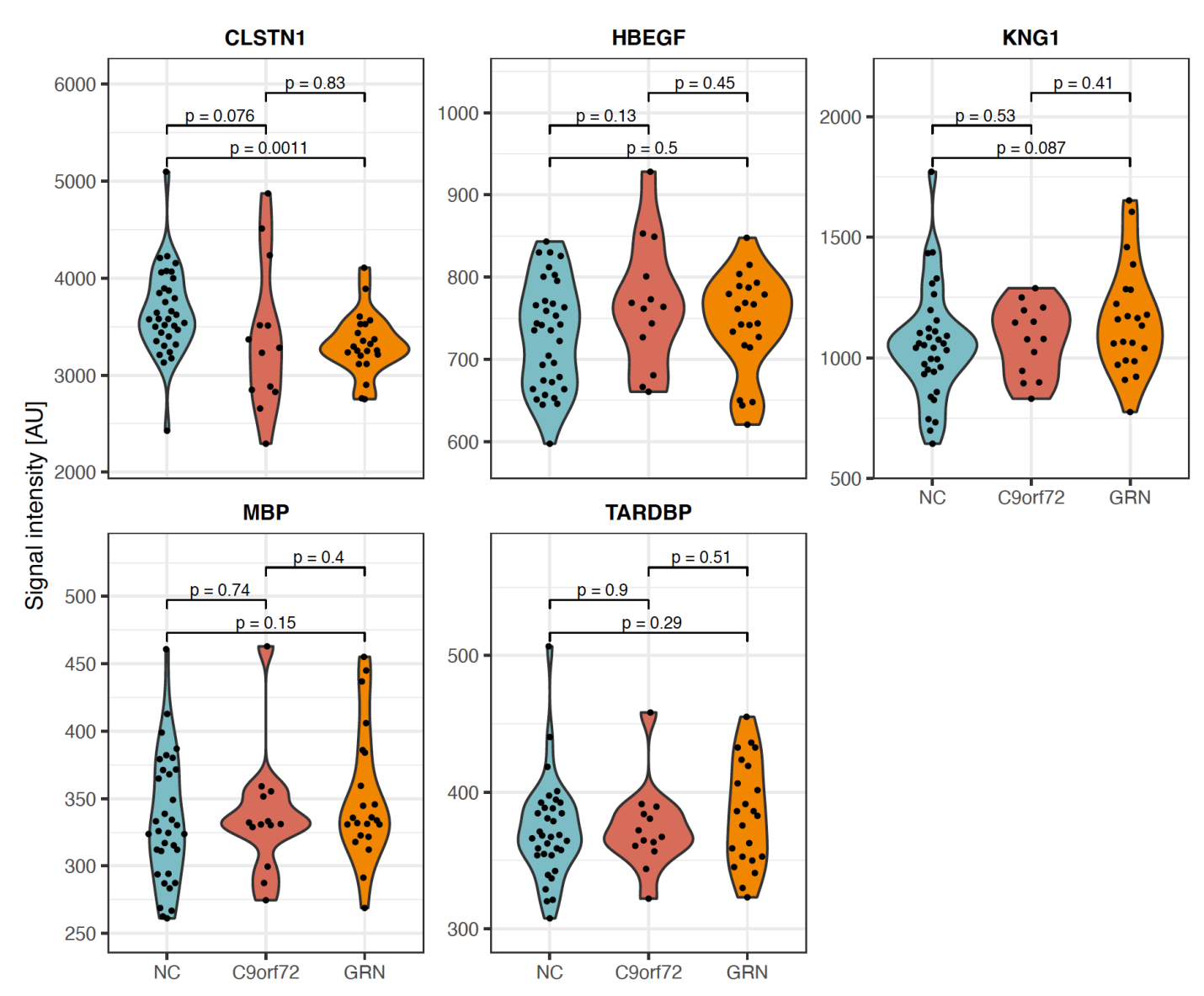
*

## *Supplementary Figure 6: Violin plots of CLSTN1, HBEGF, KNG1, MBP and TARDBP.*


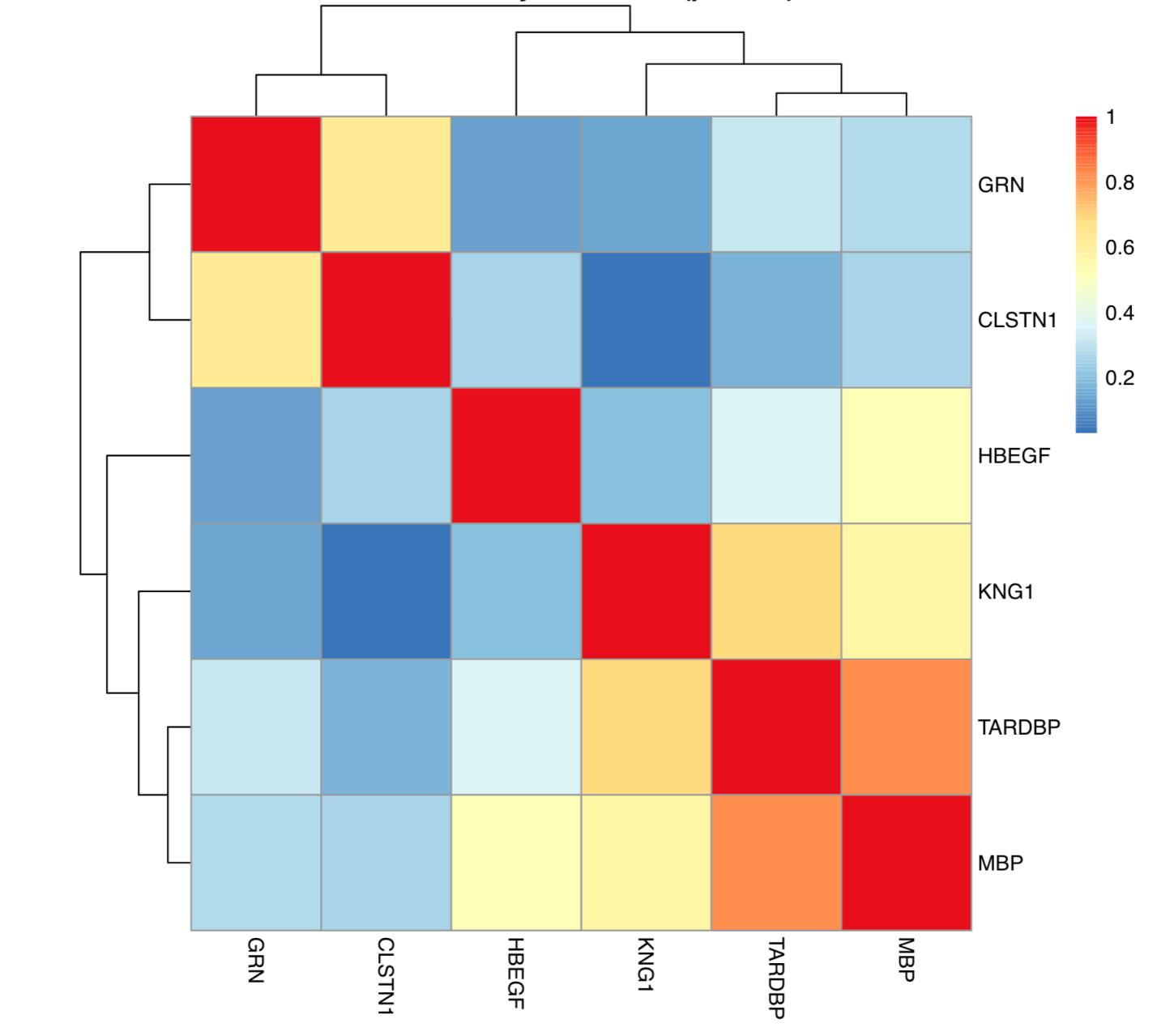


## *Supplementary Figure 7: The spearman correlation between the six proteins selected by either LASSO or Random forest when comparing PMC and NC (age and pathology filtered).*
